# CREB1/Lin28/miR-638/VASP Interactive Network Drives the Development of Breast Cancer

**DOI:** 10.7150/ijbs.36854

**Published:** 2019-10-21

**Authors:** Peng-Chao Hu, Kai Li, Yi-Hao Tian, Wen-Ting Pan, Ying Wang, Xiao-Long Xu, Yan-Qi He, Yang Gao, Lei Wei, Jing-Wei Zhang

**Affiliations:** 1 Department of Breast and Thyroid Surgery, Zhongnan Hospital, Hubei Key Laboratory of Tumor Biological Behaviors, Hubei Cancer Clinical Study Center, Wuhan University, Wuhan 430071, Hubei, China.; 2 Department of Pathology and Pathophysiology, Hubei Provincial Key Laboratory of Developmentally Originated Disease, School of Basic Medical Sciences, Wuhan University, Wuhan 430071, Hubei, China.; 3 Department of Anatomy, School of Basic Medical Sciences, Wuhan University, Wuhan 430071, Hubei, China.; 4 Department of oncology, Xiangyang No.1 People's Hospital, Hubei University of Medicine, Xiangyang 441000, Hubei, China.

**Keywords:** CREB1, Lin28, miR-638, VASP, breast cancer, cell proliferation, metastasis.

## Abstract

Breast cancer is one of the most common malignant tumors worldwide. Metastasis remains the leading cause of death in breast cancer patients. Research on the mechanism of breast cancer metastasis has become a core issue in breast cancer research. Our previous series of studies have shown that VASP, as a key oncogene, plays an important role in the development of various tumors such as breast cancer. In this study, we find that miR-638 can target to inhibit VASP expression, and Lin28 acts as an RNA-binding protein to regulate the processing of miR-638, which inhibits its maturation and promotes the expression of VASP. In addition, we also find that CREB1 acts as a transcription factor that binds to the promoter of Lin28 gene and activates the Lin28/miR-638/VASP pathway. Furthermore, CREB1 can also directly bind to the promoter of VASP, and activate VASP expression, forming a CREB/Lin28/miR-638/VASP interactive network, which plays an important role in promoting cell proliferation and migration in breast cancer. Our study explained the mechanism of CREB1/Lin28/miR-638/VASP network promoting the development of breast cancer, which further elucidated the mechanism of VASP as a key oncogene, and also provided a theoretical basis for expanding new approaches to tumor biotherapy.

## Introduction

Breast cancer is one of the most common malignant tumors among women worldwide 1. It can be divided into four subtypes based on the molecular markers (ER, PR, HER2, and Ki67) on the surface of tumor cells: Luminal A, Luminal B, HER2-enriched and Triple-negative 2. There are nearly 1.4 million new cases of breast cancer every year in the world, of which about 460,000 patients die of breast cancer and its complications 3, and its incidence is still increasing year by year, and the age of onset is younger 4. At present, the main treatment measures for breast cancer include radical surgery, chemotherapy, radiation therapy, endocrine therapy, molecular targeted therapy and immunotherapy 5. Although the 5-year survival rate of patients with early-stage breast cancer has reached more than 95% through standardized treatment, the 5-year survival rate of patients with metastatic breast cancer has not been significantly improved 6. Metastasis remains the leading cause of death in breast cancer patients 7. Therefore, research on the mechanism of breast cancer metastasis has become a core issue in breast cancer research.

Vasodilator-stimulated phosphoprotein (VASP) is an important cytoskeleton associated protein. It is located in the region of human chromosome 19q13.2-13.3, which promotes the insertion of actin monomer into the assembly end of F-actin, counteracts capping protein, and promotes F-actin assembly and prolongation 8. The biological process of skeleton rearrangement plays an important role in the occurrence and development of tumors, especially in the process of invasion and metastasis 9, 10. Studies have shown that VASP participates in the development of tumors such as gastric cancer and cervical cancer 11-15. These studies indicate that VASP is involved in the assembly and aggregation of actin filaments as a key oncogene, thereby regulating cell migration and promoting tumor invasion and metastasis. However, the current research still cannot fully explain the molecular mechanism of VASP in breast cancer, and has not established an interactive network focus on VASP. The role of VASP in breast cancer cell proliferation and migration and its regulation mechanism need further exploration.

In this study, we continue to explore the important role of the key oncogene VASP in the development of breast cancer, and explain the mechanism of the CREB1/Lin28/miR-638/VASP interactive network to promote the development of breast cancer, which further elucidate the mechanism of VASP as a key oncogene, and also provide a theoretical basis for expanding new approaches to tumor biotherapy.

## Materials and Methods

### Human breast cancer samples and Cells

Ten pairs of breast cancer tissues and their corresponding adjacent tissues were collected from Affiliated Zhongnan Hospital of Wuhan University and diagnosed by the Department of Pathology. All patients were informed and agreed. This study was carried out in accordance with the recommendations of the Ethics Board of Zhongnan Hospital with written informed consent from all subjects. All subjects gave written informed consent in accordance with the Declaration of Helsinki. The protocol was approved by the Ethics Board of Zhongnan Hospital.

Human embryonic kidney cells HEK293T (293T), normal mammary epithelial cells (MCF-10A) and the breast cancer cell (MCF-7, MDA-MD-231) were used in this study. The cell lines were purchased from the China Center for Type Culture Collection (CCTCC, Chinese Academy of Sciences, Shanghai, China). Their culture conditions are not exactly the same. MCF-10A used a special medium (DMEM/F12 medium), 5% horse serum, 20 ng/ml EGF, 100 ng/ml cholera, 0.01 mg/ml insulin, and 500 ng/mL cortisol were added. 293T, MCF-7 and MDA-MB-231 were cultured in DMEM medium, and this cell culture medium was included 10% fetal bovine serum (FBS) and 1% penicillin and streptomycin. The cells were cultured in an incubator (Esco, Singapore) with 5% CO^2^, 37 °C.

### Plasmids, siRNA and cell transfection

The Lin28 overexpression plasmid was bought from Era Biotech, and the plasmids was validated by sequencing. Expression plasmid of miR-638 was constructed by PCR using human genomic DNA as a template. PCR product (containing pre-miRNA) was digested with BamHI/XhoI and ligated into pcDNA6.2-GW/EmGFP-miR vector (Invitrogen) to yield pcDNA6.2-miR-638 which was confirmed by DNA sequencing. Three siRNA sequences of the target gene VASP and CREB were designed respectively (Shanghai GKS), and then synthesized the interference plasmid separately. pLKO-GFP-siVASP1 (target sequence: AAGGAGGTGGGCCCCTCC), pLKO-GFP-siVASP2 (target sequence: ACGGGTCCCCAGGCCTTCA), pLKO-GFP-siVASP3 (target sequence: AGCCGGACTCTATGGGGCG), pLKO-GFP-siLin28A (target sequence: CTACAACTGTGGAGGTCTA), pLKO-GFP-siLin28B (target sequence: CTCCTTCTTTTAGGCTTCTAA), pLKO-GFP-siTUT4 (target sequence: GCAGCTATTGATCCTAGAG), and control plasmid pLKO-GFP-siCon (control sequence: GCAAGCTGACCCTGAAGTT). After plasmids were transfected into 293T and MCF-7 cells by Lipofectamine 2000 transfection reagent, qPCR was used to detect the interference efficiency. The lentivirus vector was constructed with the highest interference efficiency sequence. Then cells with the highest interference efficiency were selected, passaged and saved. The primers of plasmids construction can be seen in **Supplementary Table 1**.

### Reverse transcription and quantitative polymerase chain reaction (RT-qPCR)

The mRNA expression level of gene was examined by qPCR (SYBR Green Supermix, Biorad, China) normalized to expression of GAPDH. First, Total RNA was extracted from cells using Trizol reagent (Applied Biosystem Inc., USA according to the manufacturer's protocol. The cDNA was obtained by RevertAid^TM^ First Strand cDNA Synthesis Kit (Fermentas, Canada). Then the expression level of target genes was analyzed by cDNA. Mixed 2×SYBR Green PCR Master Mix 5 µl, forward and reverse primers 2 µl, appropriate amount of cDNA, and ddH_2_O to 10µl volume. qPCR conditions consisted of the following: 95 ˚C for 3 min for denaturation; 95 ˚C for 20 sec for annealing; and 72 ˚C for 5 min for extension, for 40 cycles. The relative expression of each gene was calculated by using the 2^-∆∆Ct^ method. Each experiment was repeated three times. The primers of qPCR can be seen in **supplementary Table 1**.

### Western blot

Cells were collected, and the protein was extracted with RIPA lysate. Then the protein was denatured by boiling at 99°C for 5 min, and quantified with BCA Protein Assay Kit (Beyotime Biotechnology Co, Jiangsu, China). After SDS-PAGE electrophoresis and transmembrane, the protein was binded to PVDF membrane, then blocked the nonspecific sites with 5% skimmed milk powder. Meanwhile, a primary antibody, including CREB1 (dilution of 1:1000, Cell signaling Technology, USA), p-CREB1 (dilution of 1:1000, Cell signaling Technology, USA), VASP (dilution of 1:1000, Cell signaling Technology, USA), HA (dilution of 1:1000, Cell signaling Technology, USA), GAPDH (dilution of 1:5000, Proteintech, USA), should be prepared, and then incubating the PVDF membrane overnight at 4°C. The membrane was incubated with the corresponding secondary antibody horseradish peroxidase (dilution of 1:1000, Proteintech, USA) for 1 h at room temperature, and finally detected by ECL reagents (Tanon, Shanghai, China). The optical density of bands was measured by a computer-assisted imaging analysis system (Tanon, Shanghai, China) and the relative protein expression levels were normalized to GAPDH.

### Immunofluorescence

Immunofluorescence technique was a common method for detecting the morphology and distribution of intracellular proteins. The technique was used to detect the distribution of VASP and actin in breast cancer cells. Stable cells with GFP green fluorescent labeling in logarithmic growth period were inoculated on circular glass slides and treated in groups. The morphology and distribution of F-actin can be observed by fluorescence microscope (Olympus, Japan) or confocal laser microscope (Leica, Germany) to detect the distribution of immunoreaction with red fluorescent Cy3 labeled antibody or phalloidin (YEASEN, Shanghai).

### Cell proliferation assay

Colony formation assay were used to detect the cell proliferation. Cells (2 × 10^2^ cells/well) were evenly seeded in 6-well plates, transfected with a specific plasmid and cultured. About one week later, the paraformaldehyde solution immobilized the cells. Stain with crystal violet solution and scan the results under a microscope.

### Wound healing assay

Wound healing assay can be used to detect the ability of cell migrate. In this experiment, it was used to detect changes in the migration ability of breast cancer cell lines after specific genes were stably knocked down. Cells (2×10^5^ cells/mL) were cultured in a 6-well plate after specific genes were stably knocked down; meanwhile a set of negative controls was set up. When the cells reached 80% to 90% confluence, cells were scratched. And the cells were continued to culture for 0, 24, and 48 h in the serum-free medium. Then cells were fixed and photographed under a light microscope (Olympus, Japan), the ability of the cells to migrate was determined by calculated the number of cells that migrated to the scratched area.

### Transwell assay

Some 24-well plates and a polyvinyl-pyrrolidone-free polycarbonate filter (8 µm pore size) were needed in the experiment. When the transwell assay was used to detect the ability cell invasion, the 8 microspore was coated with Matrigel Matrix (BD, USA). Cells that specific genes were stably knocked down, at a density of 1 × 10^5^ in 100 μL serum-free medium, and the lower chamber contained 700 μL of culture medium with 20% FBS, a set of negative controls also was set up. After cells were cultured for 20 h, cells were fixed, stained, and counted the cell number in the surface of the lower chamber under light microscope (Olympus, Japan).

### Nude mouse orthotopic transplantation tumor models

The tumorigenic ability of breast cancer cells after VASP knockout was observed by orthotopic transplanted tumor model in nude mice. A nude mouse model of orthotopic transplanted tumor was established by subcutaneous injection of the second breast pad (1 × 10^8^ cell/mL) in 4-week-old female Balb/c nude mice (purchased from the animal research center of Wuhan University/ABSL-III laboratory). Then the tumor growth rate and final tumor volume and mass were observed and recorded. What's more, the metastasis of breast cancer in mice was analyzed by in-vivo imaging system. Our study was approved by the Ethics Board of School of Basic Medical Sciences, Wuhan University.

### Chromatin immunoprecipitation (ChIP)

ChIP can be used to detect protein-DNA interactions. The protein-DNA complex was immobilized in a living cell state and randomly cut into small chromatin fragments in a certain length. The complex was precipitated by immunological method, and the DNA fragment of the target protein binding was specifically enriched. The DNA was purified by PCR cleanup kit (Axygen, USA), the concentration of DNA was determined and the DNA information of protein interaction was obtained. The primers of ChIP qPCR can be seen in supplementary Table 1.

### Luciferase assay

Based on the predicted binding site of CREB and the promoter of VASP using JASPAR, we constructed wild-type and mutant plasmids carrying different lengths of the VASP promoter including pGL3-VASP(-1870~+230), pGL3-VASP(-1312~+230), pGL3-VASP(-1312~+230), and pGL3-VASP842mut (-1312~+230). pGL3-Basic (Promega, Madison, WI, USA) acts as the blank control. A fragment of the VASP mRNA-3'UTR was amplified from cDNA by PCR and cloned into pMIR-REPORT vector (ThermoFisher) to make pMIR-VASP-3' UTR construct. The miR-638 binding site mutation reporter plasmid (pMIR-VASP-3' UTR-Mut) was constructed by site-directed mutation PCR and was validated by sequencing. Cells were seed in 24-well plates and co-transfected with the mixture containing 100 ng of pMIR-VASP-3' UTR or pMIR-VASP-3' UTR-Mut, 400 ng of pcDNA6.2-miR-638 or pcDNA6.2-Control, and 10 ng of pRL-TK using Lipofectamine 2000 (Invitrogen). Cells were harvested 48 hours after transfection.The activity of firefly luciferase and Renilla luciferase was detected by the Dual Luciferase Reporter Assay System (Promega, Wisconsin, USA). The value of firefly luciferase was standardized with the corresponding Renilla luciferase activity.

### Statistical analysis

Statistical analysis was performed using software SPSS. Each set of experiment was repeated 3 times. The data was expressed as the mean ± standard deviation (SD). The significance of differences between CREB1 and VASP expression was detected by Pearson's correlation test, the *in vitro* data using Student's t test (two-tailed), and the *in vivo* data using the Mann-Whitney U test. p<0.05 was considered statistically significant.

## Results

### VASP is highly overexpressed in breast cancer cells and tissues and is significantly associated with poor prognosis in breast cancer patients

**Figure 1:**
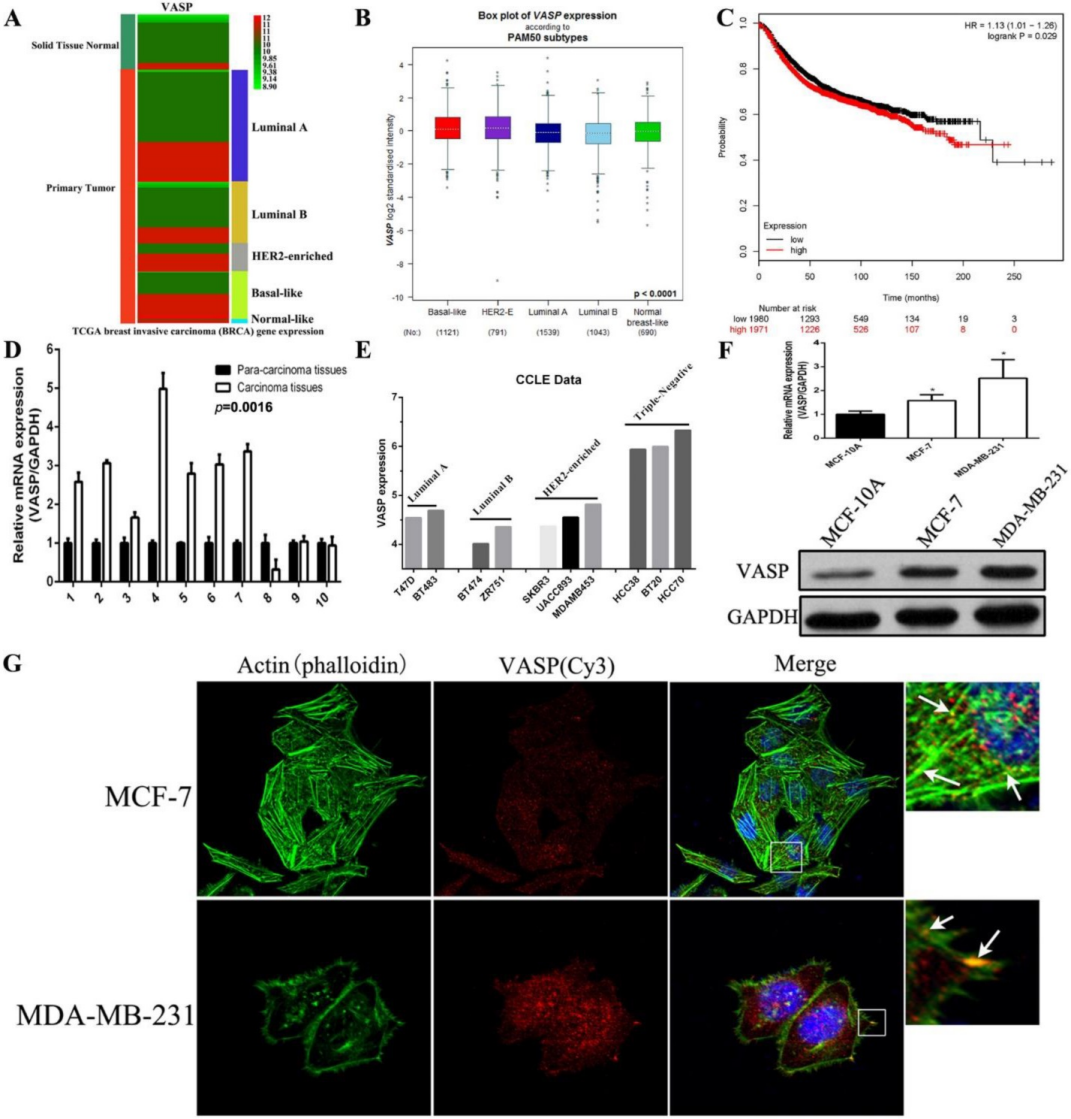
** VASP is highly overexpressed in breast cancer cells and tissues and is significantly associated with poor prognosis in breast cancer patients.** (A) The expression levels of VASP between normal breast tissue and breast cancer tissue were analyzed using the TCGA database. (B) The expression of VASP in normal breast tissue and invasive breast cancer tissues was analyzed by the Breast Cancer Gene-Expression Miner v4.2 database. (C) The correlation between the expression level of VASP and the survival of breast cancer patients were analyzed using Kaplan-Meier plotter database. (D) VASP mRNA expression was detected in 10 pairs of breast cancer tissues and their corresponding adjacent tissues by qPCR. (E) The expression of VASP in different cell lines was analyzed using the CCLE database. (F) The mRNA and protein expression levels of VASP in breast cancer cells MCF-7, MDA-MB-231 and normal breast cell MCF-10A were detected by qPCR analysis and western blot. (G) The fluorescence intensity of VASP and cytoskeleton actin filaments was detected by immunofluorescence assay. Values represent the mean ± SD from three independent measurements. *P < 0.05.

We first analyzed the expression of VASP in normal breast tissues and different sub-types of breast cancer tissues using the BRCA (breast invasive carcinoma) data from TCGA. The results showed that the expression level of VASP in breast cancer tissues was significantly higher than that in normal breast tissues, and the expression level of VASP in HER2-Enriched and Basal-like breast cancer was higher than that in luminal A/B breast cancer **(Figure 1A)**. The expression of VASP within each sub-type of breast cancer tissues was also analyzed by the Breast Cancer Gene-Expression Miner v4.2 database. The results were similar with TCGA database, the VASP expression in Basal-like and HER2-Enriched breast cancer with higher malignancy was slightly higher than in luminal A and luminal B breast cancer **(Figure 1B)**. In addition, Kaplan-Meier plotter database analysis showed a negative correlation between the expression level of VASP and the survival of breast cancer patients (P=0.029) **(Figure 1C)**. To further validate this result, we collected 10 pairs of breast cancer tissues and their corresponding adjacent tissues. The results of qPCR showed that compared to adjacent tissues, VASP mRNA expression were significantly higher in 7 cases breast cancer samples (7/10) **(Figure 1D)**. Furthermore, we analyzed VASP expression in a variety of breast cancer cell lines using the CCLE database, the results showed that the VASP expression in triple negative breast cancer cell lines was significantly higher than in luminal cancer cell lines **(Figure 1E)**. The mRNA and protein levels of VASP was detected in normal breast cell MCF-10A, luminal A breast cancer cell MCF-7 and triple negative breast cancer cell MDA-MB-231. The VASP mRNA and protein levels in MCF-7 and MDA-MB-231 were significantly higher than those in MCF-10A, and the expression level was higher in MDA-MB-231 with higher malignancy and stronger invasive ability than MCF-7 cells with lower malignancy and weaker invasive ability **(Figure 1F)**. The results of immunofluorescence assay also showed that the fluorescence intensity of VASP in MDA-MB-231 cells was significantly higher than that of MCF-7, and VASP was distributed along the cytoskeleton actin filaments and clustered at the ends of the fibers **(Figure 1G)**. In summary, VASP is overexpressed in breast cancer cells and tissues, positively correlated with tumor malignancy, and significantly associated with poor prognosis in breast cancer patients.

### Knockdown of VASP can inhibit breast cancer cell proliferation, migration and tumor growth and metastasis

**Figure 2:**
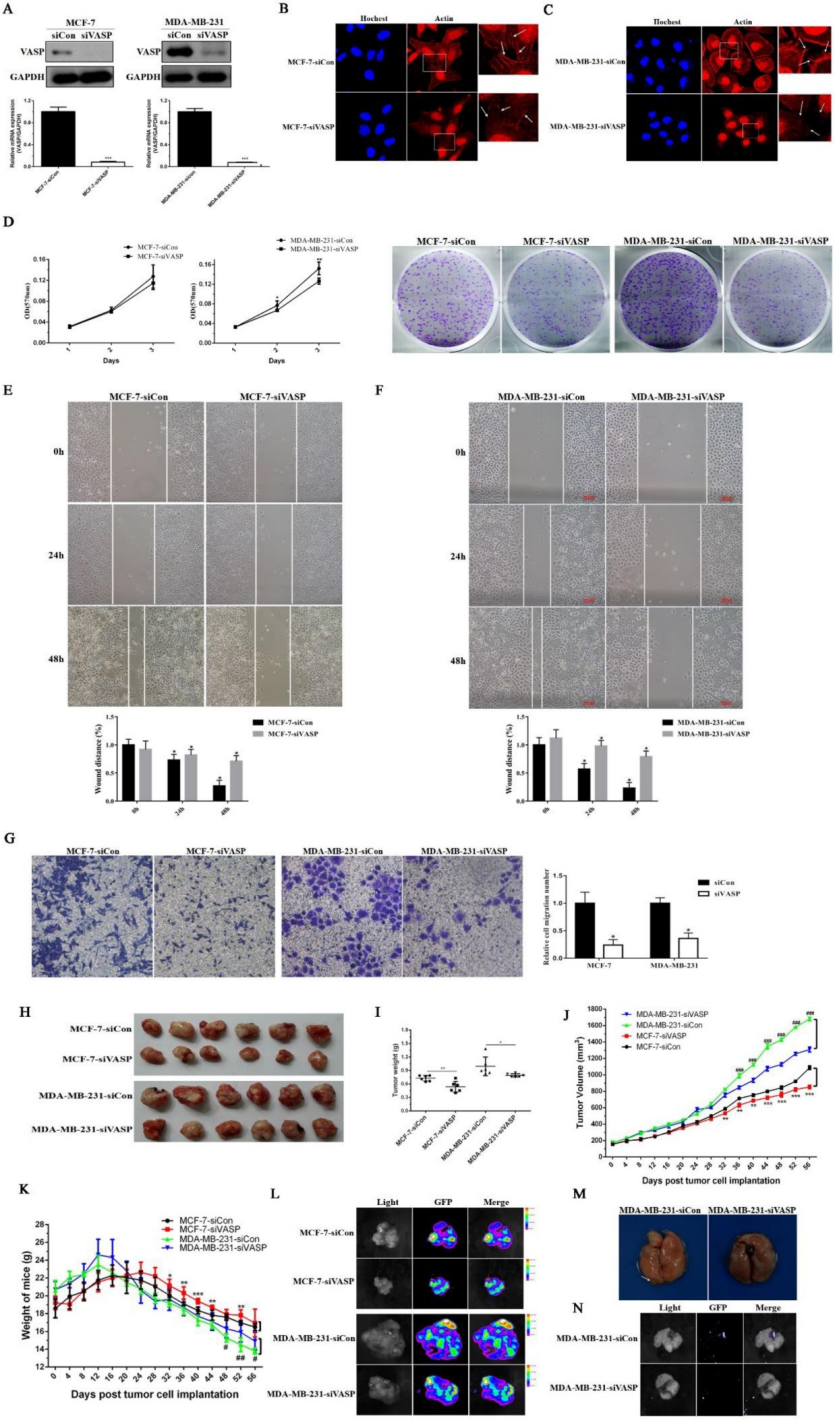
** Knockdown of VASP can inhibit breast cancer cell proliferation, migration and tumor growth and metastasis.** (A) VASP knockdown stable cell lines were constructed in MCF-7 and MDA-MB-231 cells, which were detected by western blot and qPCR. Knockdown of VASP significantly affected normal aggregation of F-actin in breast cancer cells MCF-7 (B) and MDA-MB-231 (C). (D) Knockdown of VASP can significantly inhibit the proliferation of breast cancer cells MCF-7 and MDA-MB-231. Knockdown of VASP significantly inhibited the migration of breast cancer cells MCF-7 (E) and MDA-MB-231 (F), which were detected by wound healing assay. (G) Knockdown of VASP significantly inhibited the migration of breast cancer cells MCF-7 and MDA-MB-231, which were detected by Transwell assay. (H) The effect of VASP knockdown in MCF-7 and MDA-MB-231 cells on the growth of xenografts in nude mice. (I) The effect of VASP knockdown in MCF-7 and MDA-MB-231 cells on the weight of xenografts in nude mice. (J) The effect of VASP knockdown on the volume of xenografts in nude mice after tumor cells implantation, * means MCF-7-Con vs MCF-7-siVASP, # means MDA-MB-231-siCon vs MDA-MB-231-siVASP. (K) The effect of VASP knockdown on the body weight of the mice after tumor cells implantation, * means MCF-7-Con vs MCF-7-siVASP, # means MDA-MB-231-siCon vs MDA-MB-231-siVASP. (L) The transplanted tumors of MCF-7 and MDA-MB-231 were detected by small animal live imaging. (M) The nude mice were sacrificed, and the lungs were collected and used to detect the tumor metastasis (the arrow indicated the tumor metastasis in the lung). (N) The tumor metastasis in the lung was detected by small animal live imaging. Values represent the mean ± SD from three independent measurements. *, # P < 0.05; **, ## P < 0.01; ***, ### P < 0.001.

In view of the higher expression of VASP in breast cancer cell lines and breast cancer tissues, the expression level of VASP was negatively correlated with the prognosis of breast cancer patients, suggesting that VASP was closely related to the development of breast cancer. Next, we constructed the VASP knockdown stable cell lines in MCF-7, MDA-MB-231 cells. The mRNA and protein expressions of VASP in MCF-7-siVASP, MCF-7-sicon and MDA-MB-231-siVASP and MDA-MB-231-sicon were detected by qPCR and Western blot. The results showed that the mRNA and protein levels of VASP were significantly decreased, which demonstrated that the stable cell lines were successfully constructed **(Figure 2A)**.

Immunofluorescence staining was used to further detect the distribution of actin fiber F-actin in VASP knockdown breast cancer cell lines. The results showed that knockdown of VASP significantly affected normal aggregation of F-actin in breast cancer cells MCF-7 and MDA-MB-231 **(Figure 2B-C)**. Using MTT and plate colony formation assays, we studied the proliferative capacity of VASP knockdown cell lines. Compared to the control group, knockdown of VASP can significantly inhibit the viability and colony forming ability of breast cancer cells MCF-7 and MDA-MB-231 **(Figure 2D)**. The results of wound healing assay showed that knockdown of VASP significantly inhibited the migration of breast cancer cells MCF-7 and MDA-MB-231 **(Figure 2E-F)**. The results of transwell assay were consistent with wound healing assay **(Figure 2G)**. In addition, we observed the effects of VASP knockdown on the growth and metastasis of breast cancer cells by nude mice tumor-bearing experiments. It was found that knockdown of VASP can significantly slow down the growth rate of the tumor and ultimately reduce the weight and volume of the tumor **(Figure 2H-J)**. As the tumor burden increased, the body weight of the mice decreased significantly, showing a cachexia state and knockdown of VASP can significantly improve the living conditions of tumor-bearing mice **(Figure 2K)**. At the same time, we also carried out small animal live imaging to observe the lung metastasis of breast cancer cells. The results showed green fluorescence in the transplanted tumor, indicating that the stable tumor cell transplanted tumor model was successfully established **(Figure 2L)**. In the control group of MDA-MB-231 tumors, we found tumor metastasis in the lungs of nude mice **(Figure 2M)**, which showed green fluorescence **(Figure 2N)**, but no metastases were found in the VASP knockdown group. Although the incidence of lung metastasis was not high (3/8), it suggested that the invasion and metastasis ability of breast cancer cells decreased after VASP knockdown. Taken together, the above results indicate that VASP knockdown can inhibit proliferation, invasion and metastasis of breast cancer cells.

### CREB promotes the expression of VASP at the transcriptional level, regulating breast cancer cell proliferation

**Figure 3:**
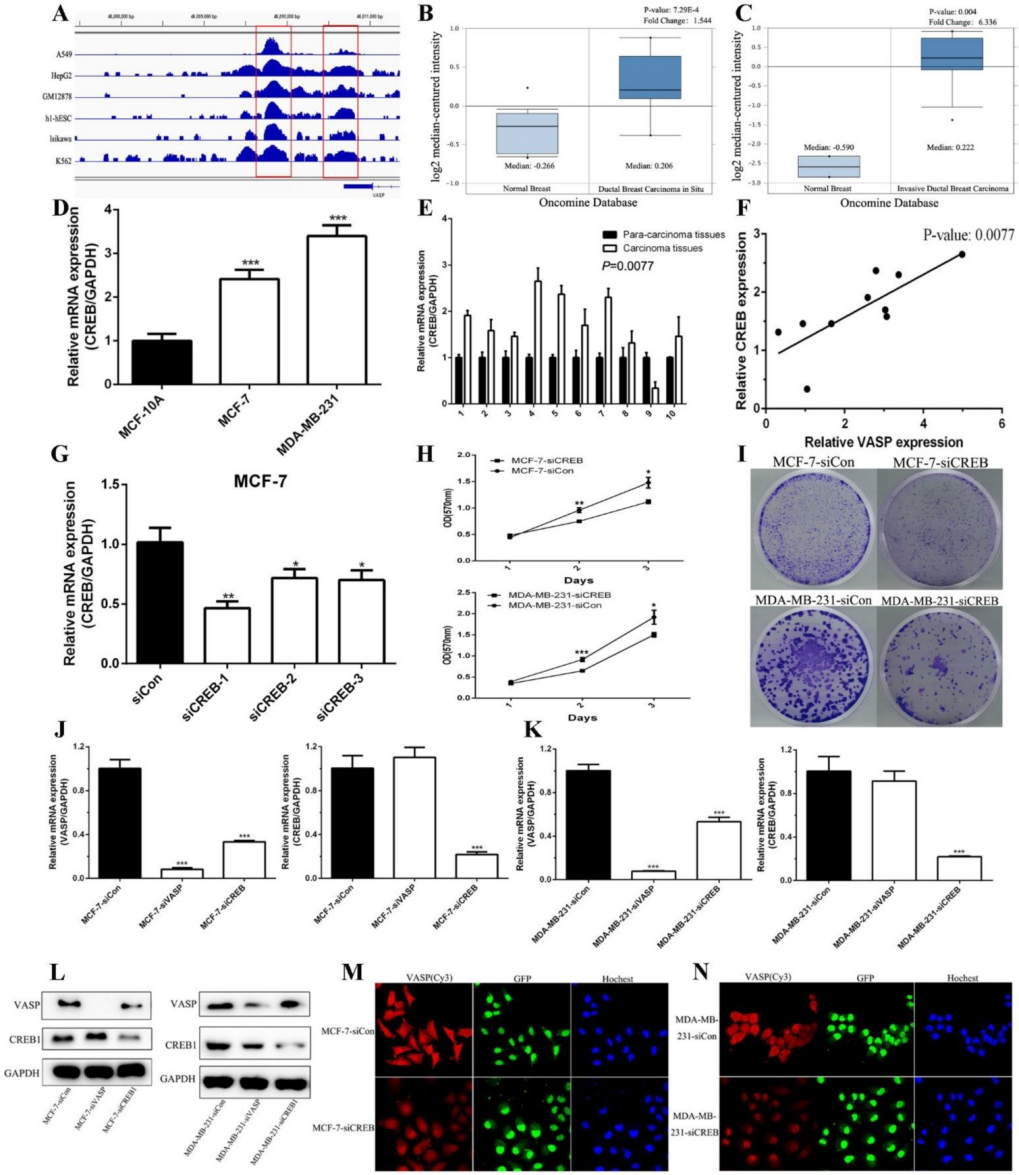
** CREB1 is upregulation in breast cancer and closely related to VASP.** (A) CREB1 is significantly enriched in the promoter region of VASP, which was analyzed by ChIP-Seq data in the GEO database. The expression of CREB1 in ductal breast carcinoma *in situ* (B) and invasive ductal breast carcinoma tissues (C) were analyzed using the Oncomine Database. (D) The mRNA expression levels of CREB1 in breast cancer cells MCF-7, MDA-MB-231 and normal breast cell MCF-10A were detected by qPCR analysis. (E) CREB1 mRNA expression was detected in 10 pairs of breast cancer tissues and their corresponding adjacent tissues by qPCR. (F) The correlation between CREB1 and VASP mRNA expression was detected in 10 pairs of breast cancer tissues and their corresponding adjacent tissues using Pearson's correlation test. The siRNAs of CREB1 were synthesized and transfected into MCF-7 (G) cells to detect the efficiency of knockdown. The effect of CREB1 knockdown on the proliferation of breast cancer cells MCF-7 and MDA-MB-231 was detected by MTT (H) and colony formation assay (I). The effect of VASP or CREB knockdown on the mRNA expression of VASP and CREB was detected by qPCR in MCF-7 (J) and MDA-MB-231 cells (K). (L)The effect of VASP or CREB knockdown on the protein expression of VASP and CREB was detected by western blot in MCF-7 and MDA-MB-231 cells. The effect of CREB knockdown on the protein expression of VASP was detected by immunofluorescence staining in MCF-7 (M) and MDA-MB-231 cells (N). Values represent the mean ± SD from three independent measurements. *P < 0.05, **P < 0.01, ***P < 0.001.

The results of the increasing expression of VASP mRNA levels indicated that transcriptional activation may participate in the regulation mechanism of VASP in breast cancer. The software MatInspector Professional (Genomatix, JASPAR, TRANSFAC) was used to search potential transcription factor binding sites in the VASP promoter. Three CREB1 potential binding sites (-1538bp, -1130bp, and -840bp) were identified within a 2-kb region upstream of the promoter region of VASP. We analyzed the CREB1-related ChIP-Seq data in the GEO database, which showed that CREB1 was significantly enriched in the promoter region of VASP** (Figure 3A)**. These results suggested that CREB1 may regulate the expression of VASP as a transcription factor. We analyzed the Oncomine Database and found that the expression of CREB1 in ductal breast carcinoma *in situ* and invasive ductal breast carcinoma tissues were significantly higher than that in normal breast tissues** (Figure 3B-C)**. To further demonstrate the results, we examined the expression of CREB1 mRNA in breast cancer tissues and cell lines by qPCR. The results showed that the expression levels of CREB1 in tumor cell lines and breast cancer tissues were significantly higher than that of normal cell lines and breast tissues **(Figure 3D-E)**. Correlation analysis revealed that there was a significant positive correlation between CREB1 and VASP expression **(Figure 3F)**. To further clarify the relationship between CREB1 and VASP, we synthesized siRNA to knock down CREB1 **(Figure 3G)**. MTT and Colony formation assays showed that knockdown of CREB1 significantly inhibited the proliferative capacity of breast cancer cells MCF-7 and MDA-MB-231 **(Figure 3H-I)**. In MCF-7 and MDA-MB-231 cells, qPCR, western blot and immunofluorescence staining showed that knockdown of CREB1 significantly inhibited the mRNA and protein expression levels of VASP **(Figure 3J-N)**.

**Figure 4:**
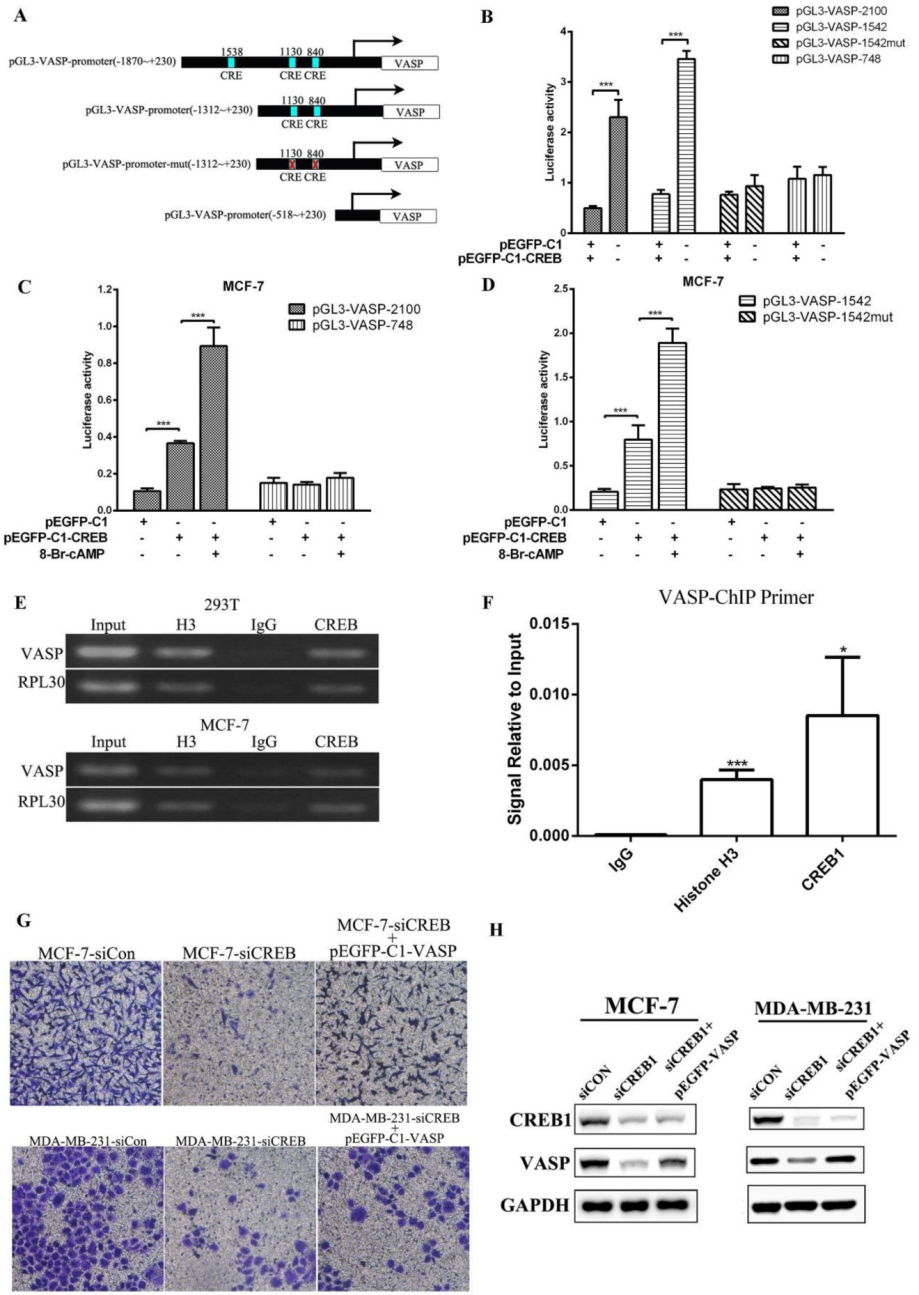
** CREB promotes the expression of VASP at the transcriptional level, regulating breast cancer cell migration.** (A) Bioinformatics analysis showed that -840, -1130 and -1538 sites of VASP promoter were the potential binding sites of CREB1. And reporter gene plasmids of different lengths promoter (pGL3-VASP-748, pGL3-VASP-1542, pGL3-VASP-2100), and CRE site-mutated reporter plasmids (pGL3-VASP-1542-mut) were constructed. (B) The effect of CREB1 overexpression on the activity of pGL3-VASP-748, pGL3-VASP-1542, pGL3-VASP-2100 and pGL3-VASP-1542-mut was detected by Luciferase assay. (C) The effect of 8-br-cAMP treatment combined with CREB1 overexpression on the activity of pGL3-VASP-748 and pGL3-VASP-2100 was detected by Luciferase assay. (D) The effect of 8-br-cAMP treatment combined with CREB1 overexpression on the activity of pGL3-VASP-1542 and pGL3-VASP-1542-mut 1 was detected by Luciferase assay. CREB1 can bind to promoter region of VASP, which was detected by ChIP (E) and ChIP-qPCR (F). The effect of CREB1 knockdown on the migration of breast cancer cells MCF-7 and MDA-MB-231 (G) was detected by Transwell assay. (H) The protein expression of VASP and CREB was detected by western blot in MCF-7 and MDA-MB-231 cells. Values represent the mean ± SD from three independent measurements. *P < 0.05, **P < 0.01, ***P < 0.001.

To confirm the positive transcriptional regulation of VASP expression by CREB1, we performed luciferase assays. We designed reporter gene plasmids of different lengths promoter (pGL3-VASP-748, pGL3-VASP-1542, pGL3-VASP-2100), and CRE site-mutated reporter plasmids (pGL3-VASP-1542-mut) **(Figure 4A)**. Luciferase assay results showed that CREB1 overexpression significantly enhanced the activity of pGL3-VASP-1542 and pGL3-VASP-2100, but had no significant effect on the activity of pGL3-VASP-1542-mut in 293T cells **(Figure 4B)**. 8-br-cAMP can act as an agonist of CREB1, and 8-br-cAMP treatment can significantly increase the activation of pGL3-VASP-2100 and pGL3-VASP-1542 by CREB1 overexpression, while had no effect on pGL3-VASP-1542-mut **(Figure 4C-D)**. These results showed that -840 and -1130 sites of VASP promoter were the key binding sites of CREB1 to VASP promoter. ChIP-PCR assays then were used to verify that CREB1 physically interacts with these predicted binding sites on VASP promoter. The fragments containing CREB1 binding motifs could be detected in CREB1 immunoprecipitated DNA fragments but not in IgG immunoprecipitated controls** (Figure 4E-F)**. In MCF-7 and MDA-MB-231 cells, VASP overexpression significantly reversed the inhibitory effect of CREB1 knockdown on the migration of breast cancer cells **(Figure 4G-H)**. These results indicate that CREB promotes the expression VASP at the transcriptional level and thus regulates breast cancer cell proliferation and migration.

### miR-638 can target VASP in breast cancer

**Figure 5:**
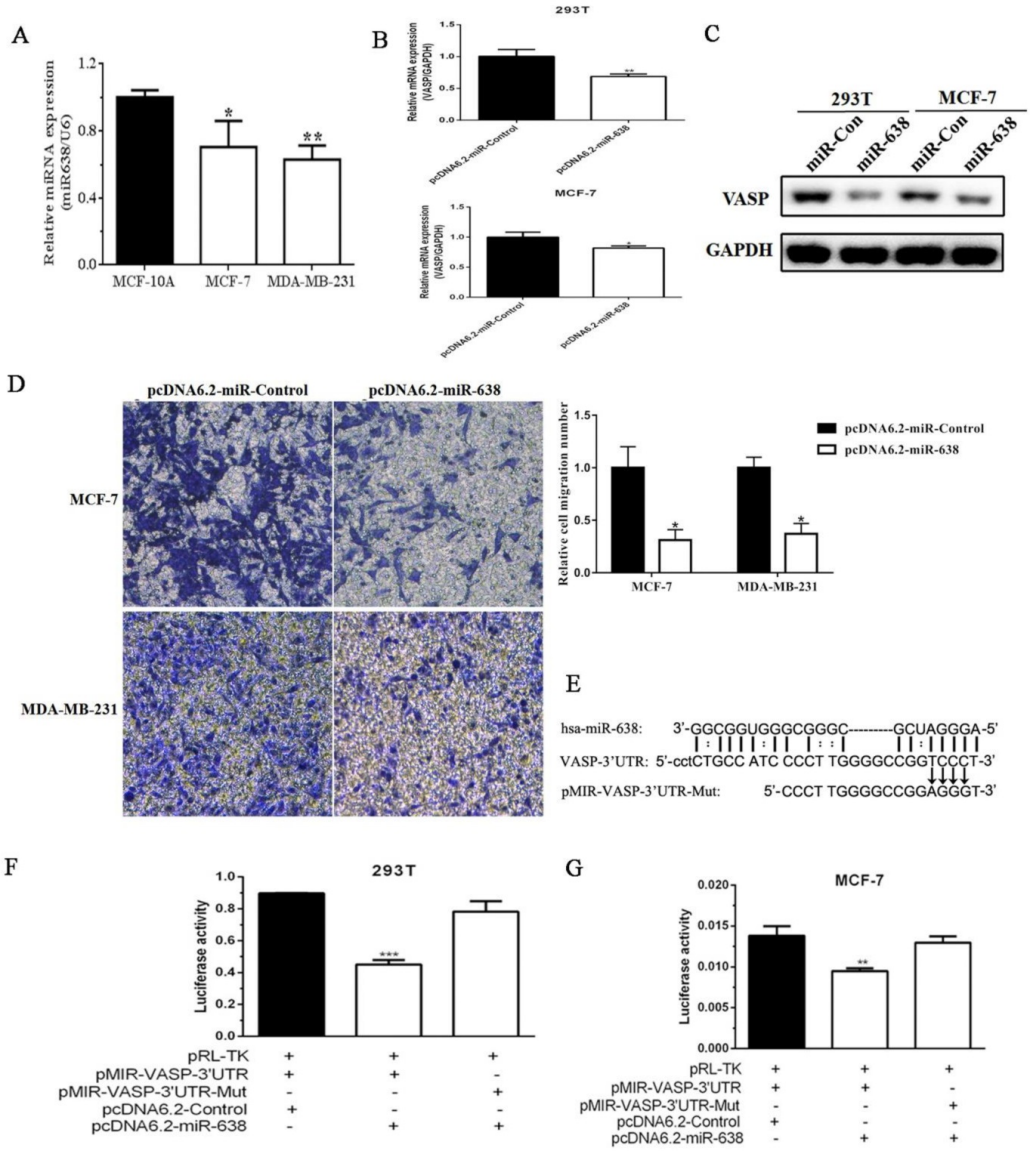
** miR-638 can target VASP in breast cancer.** (A) The expression levels of miR-638 in MCF-10A, MCF-7 and MDA-MB-231, which was detected by qPCR. (B) The effect of miR-638 overexpression on the mRNA expression of VASP in 293T and MCF-7 cells. (C) The effect of miR-638 overexpression on the protein expression of VASP in 293T and MCF-7 cells. (D) The effect of miR-638 on the migration of breast cancer cells MCF-7 and MDA-MB-231. (E) The reporter gene plasmid containing the VASP 3' UTR region (pMIR-VASP-3' UTR) and miR-638 binding site mutation reporter plasmid (pMIR-VASP-3' UTR-Mut) were constructed. The effect of miR-638 on the activity of pMIR-VASP-3' UTR or pMIR-VASP-3' UTR-Mut reporter gene in 293T (F) and MCF-7 (G) cells, which were detected by the Luciferase assay. Values represent the mean ± SD from three independent measurements. *P < 0.05, **P < 0.01, ***P < 0.001.

In addition to transcriptional regulation, the post-translational regulation also played an important role in the gene expression. Given that miRNAs emerged as powerful regulators of gene expression and were involved in cancer progression, and we found that some miRNAs were downregulated in breast cancer tissues by miRNA expression profiling in our previous study (data was not shown), so we decided to screen miRNAs that regulate VASP expression and function. Next, we examined the expression levels of miR-638 in MCF-10A, MCF-7 and MDA-MB-231 by qPCR. The results showed that the expression level of miR-638 in MCF-7 was higher than that of MDA-MB-231, but both were significantly lower than MCF-10A **(Figure 5A)**. The results suggested that miR-638 was downregulated in breast cancer cells, and its expression trend was negatively correlated with VASP. To examine the effect of miR-638 on VASP mRNA and protein expression, we constructed the miR-638 overexpression plasmid pcDNA6.2-GW/EmGFP-miR-638 (pcDNA6.2-miR-638) and the control plasmid pcDNA6.2-GW/EmGFP-Control (pcDNA6.2-Control), and then transfected them into 293T and MCF-7 cells for 24 h, respectively, and the changes of VASP mRNA and protein expression were examined. The results showed that after overexpression of miR-638, the expression levels of VASP mRNA and protein in 293T and MCF-7 cells were significantly decreased **(Figure 5B-C)**, indicating that miR-638 can indeed target and inhibit VASP expression at the post-transcriptional level. In order to examine the effect of miR-638 on the migration of breast cancer cells, we overexpressed miR-638 in MCF-7 and MDA-MB-231 cells, respectively, and found that miR-638 overexpression significantly inhibited migration ability of MCF-7 and MDA-MB-231 by Transwell assay **(Figure 5D)**, which was contrary to the effect of VASP on breast cancer cells.

Using MicroCosm Target Version to predict miRNAs that may target VASP, we found that miR-638 which was downregulated in breast cancer tissues, which may bind to the 3' UTR region of VASP and play a role in inhibiting VASP expression. To validate the inhibitory effect of miR-638 on VASP at the post-transcriptional level, we constructed a reporter gene plasmid containing the VASP 3' UTR region (pMIR-VASP-3' UTR) and a miR-638 binding site mutation reporter plasmid (pMIR-VASP-3' UTR-Mut) **(Figure 5E)**. The Luciferase assay revealed that miR-638 significantly inhibited the activity of pMIR-VASP-3' UTR reporter plasmid, but had no significant effect on pMIR-VASP-3' UTR-Mut reporter plasmid in 293T and MCF-7 cells **(Figure 5F-G)**. These results indicate that miR-638 can inhibit the migration of breast cancer cells through VASP.

### Lin28 regulates the processing of miR-638, inhibits its maturation, and thus promotes the expression of VASP

Given the fact that miR-638 was down-regulated in breast cancer tissues, Next we aimed to investigate the mechanism of miR-638 expression. Biogenesis of microRNA (miRNA) involves multiple maturation steps, and there are evidences that the RNA-binding protein Lin28 plays a key role in the maturation of some miRNAs. Lin28 is a highly conserved RNA-binding protein, including two members of Lin28A and Lin28B, involved in multiple biological processes such as RNA splicing, polyadenylation, sequence editing, RNA transport, and maintenance of RNA stability and degradation, intracellular localization and translational control by recognizing and binding to the core sequence GGAG, playing an important role in the regulation of post-transcriptional gene expression 16, 17.

The analysis of sequence of pre-miR-638 indicated that pre-miR-638 contained a Lin28 binding sequence, suggesting that Lin28 may regulate the maturation process of miR-638.

**Figure 6:**
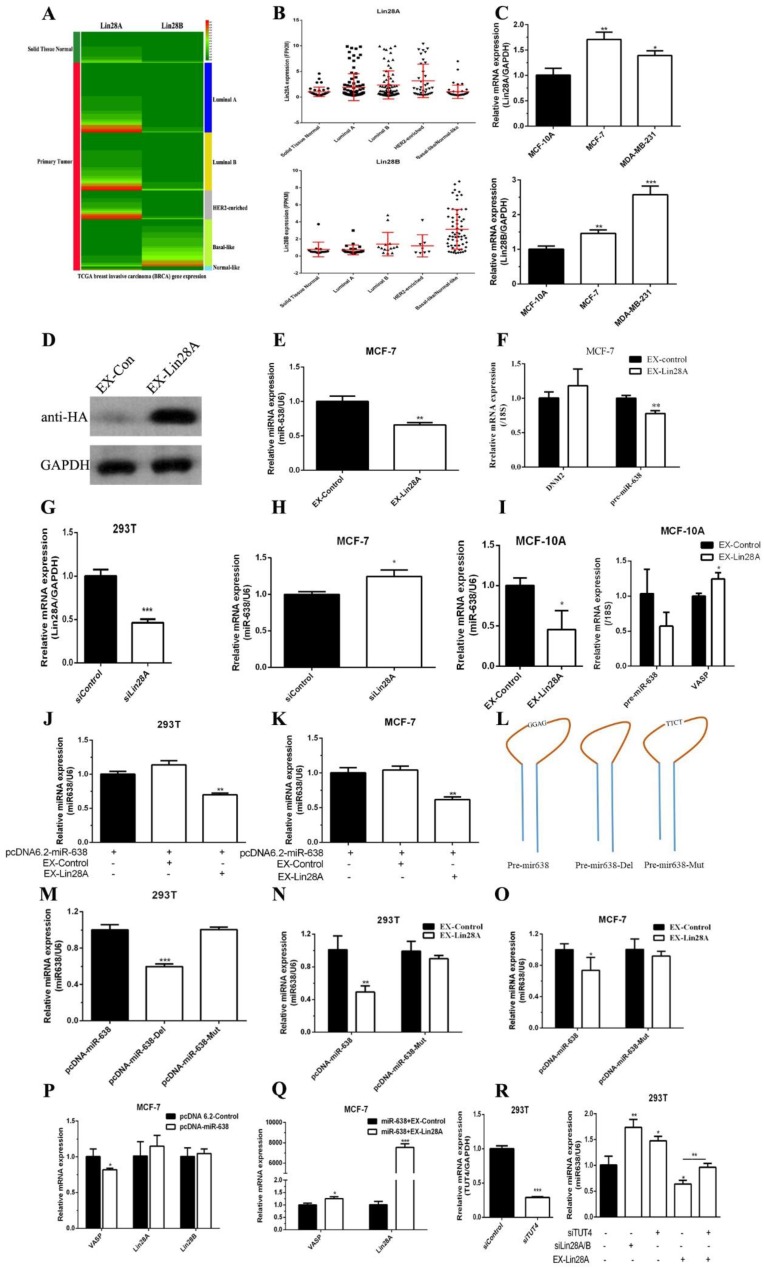
** Lin28 regulates the processing of miR-638, inhibits its maturation, and thus promotes the expression of VASP.** The differences of Lin28A and Lin28B expression between normal breast tissues and different subtype breast cancer tissues was analyzed by using the TCGA database (A-B). (C) The mRNA expression of Lin28A and Lin28B were detected by qPCR in MCF-10A, MCF-7 and MDA-MB-231 cells. (D) The overexpression of Lin28A protein was assayed by western blot. (E) Lin28A inhibited the expression of mature miR-638 in MCF-7. (F) The overexpression of Lin28A inhibited the expression of pre-miR-638 and doesn't change the expression of DNM2.** (G)** The siRNA of Lin28A was synthesized and transfected into 293T cells to detect the efficiency of knockdown. (H) The knockdown of Lin28A increased the expression of miR-638 in MCF-7. (I) The upregulation of lin28A decreased the levels of miR-638 and pre-miR-638, and increased the level of VASP in MCF-10A. The effect of Lin28A overexpression (EX-Lin28A) on the expression of miR-638 was detected by qPCR in HEK293T (J) and MCF-7 (K) cells. (L) The diagrams of wild type miR-638 (pcDNA-miR-638), deletion of the 'GGAG' in the stem region of miR-638 (pcDNA-miR-638-Del), and mutation of the 'GGAG' in the stem region of miR-638 (pcDNA-miR-638-Mut). (M) The effect of pcDNA-miR-638, pcDNA-miR-638-Del, and pcDNA-miR-638-Mut on the expression of miR-638 was detected by qPCR in 293T cells. The effect of Lin28A overexpression on the expression of pcDNA-miR-638 and pcDNA-miR-638-Mut was detected in 293T (N) and MCF-7 (O) cells. (P) The effect of miR-638 overexpression (pcDNA-miR-638) on the expression of VASP, Lin28A and Lin28B was detected by qPCR in MCF-7 cells. (Q) The effect of miR-638 overexpression (miR-638) combined with Lin28A overexpression (EX-Lin28A) on the expression of VASP and Lin28A was detected by qPCR in MCF-7 cells. (R) The effect of TUT4 knockdown (siTUT4), Lin28A/B knockdown (siLin28A/B), Lin28A overexpression (EX-Lin28A), and TUT4 knockdown combined with Lin28A overexpression on the expression of miR-638 was detected by qPCR in 293T cells.

First, we used the TCGA database to analyze the differences of Lin28A and Lin28B expression between normal breast tissues and breast cancer tissues. As shown in **Figure 6A and 6B**, the expression levels of Lin28A and Lin28B in tumor tissues were higher than those in normal breast tissues, the Lin28A expression in HER2-Enriched breast cancer was slightly higher than other sub-type breast cancers, and the Lin28B expression was significantly higher in basal-like breast cancer. The expression levels in breast cancer cells MCF-7 and MDA-MB-231 were higher than those in normal breast cells MCF-10A **(Figure 6C)**, and its expression trend was the same as VASP, and contrary to miR-638.

We also found that overexpression of Lin28A inhibited the endogenous expression of pre-miR-638 and mature miR-638 in MCF-7, which was consistent with previous reports, and the overexpression of Lin28A did not affect the expression of the host gene of miR-638, DNM2** (Figure 6D-F)** and knockdown of Lin28A in MCF-7 increased the expression of miR-638 **(Figure 6G-H)**. The upregulation of lin28A in MCF-10A decreased the levels of miR-638 and pre-miR-638. Meantime, the level of VASP mRNA had a slight increase when lin28A was overexpressed in MCF-10A **(Figure 6I)**. Lin28A overexpression also inhibited the exogenous expression of mature miR-638 in MCF-7 and HEK293T **(Figure 6J-K)**. To further validate our hypothesis, we constructed two mutant plasmids of miR-638, one of which was a key site deletion of the 'GGAG' in the stem region (pcDNA-miR-638-Del) and a key site mutation of the 'GGAG' in the stem region (pcDNA-miR-638-Mut) **(Figure 6L)**. The results showed that the key site deletion of 'GGAG' resulted in a decrease of the mature miR-638, which was consistent with the view that the length of the stem loop significantly affects miRNA maturation. The key site mutation of 'GGAG' did not significantly affect the expression level of mature miR-638 **(Figure 6M)**. However, in MCF-7 and HEK293T cells, we found that key site mutations of 'GGAG' can counteract the inhibitory effect of Lin28A on the expression level of mature miR-638 **(Figure 6N-O)**. As shown in **Figure 6P**, overexpression of miR-638 inhibited VASP mRNA expression in MCF-7 cells, but had no significant effect on mRNA expression of Lin28A and Lin28B, but in MCF-7 and HEK293T cells Lin28A can antagonize the inhibitory effect of miR-638 on VASP and promote the expression of VASP **(Figure 6Q)**. In addition, terminal uridylyltransferase 4 (TUT4) was an important cofactor for the function of Lin28. We found that knockdown of TUT4 promoted the expression of miR-638 and reversed the inhibitory effect of Lin28A on miR-638 **(Figure 6R)**. These results show that Lin28 is able to regulate the processing of miR-638 and inhibit its maturation.

### EGF increases the transcriptional level of VASP and Lin28 through phosphorylation of CREB1

**Figure 7:**
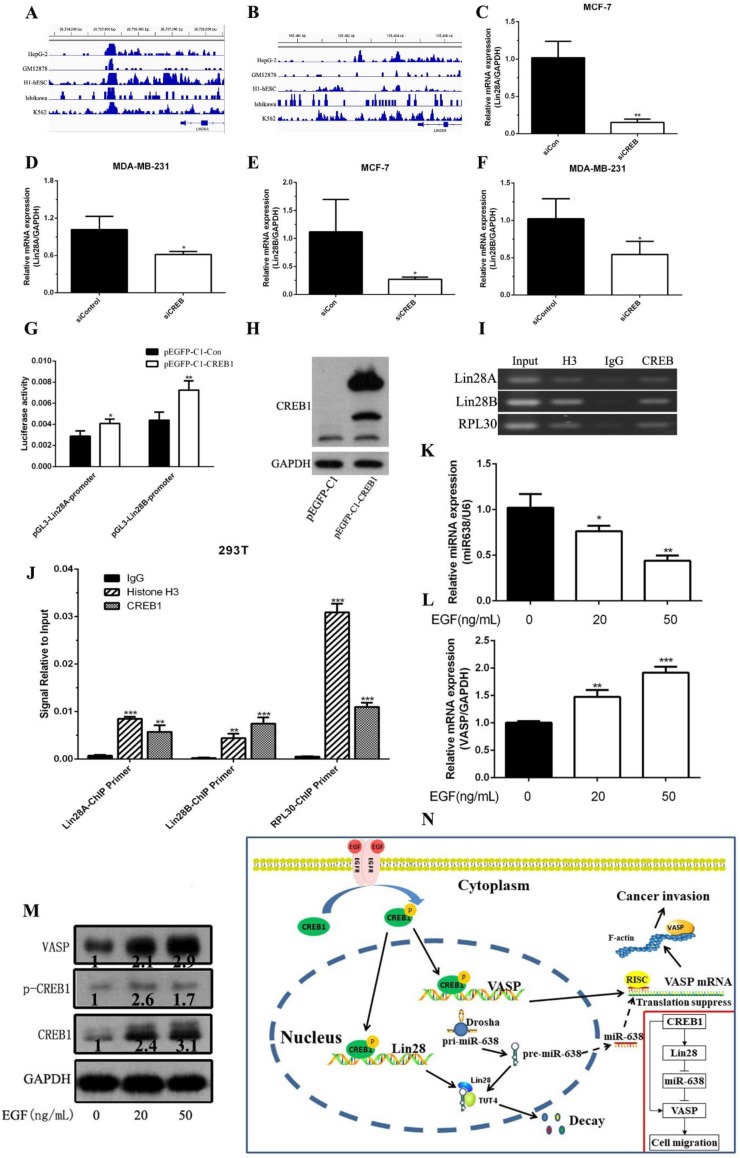
** EGF up-regulated the phosphorylation levels of CREB1 to increase the transcriptional level of Lin28.** CREB1 is enriched in the promoter region of Lin28A (A) and Lin28B (B), which was analyzed by ChIP-Seq data in the GEO database. The effect of CREB1 knockdown on the mRNA expression of Lin28A was detected by qPCR in MCF-7 (C) and MDA-MB-231 cells (D). The effect of CREB1 knockdown on the mRNA expression of Lin28A was detected by qPCR in MDA-MB-231 (E) and MDA-MB-231 cells (F). (G) The Lin28A and Lin28B promoter reporter plasmids (pGL3-Lin28A-promoter, pGL3-Lin28B-promoter) were constructed, and the effect of CREB1 overexpression on the activity of pGL3-Lin28A-promoter and pGL3-Lin28B-promoter was detected by Luciferase assay. (H) The plasmid pEGFP-CREB1 was constructed, and CREB1 overexpression was detected by western blot. CREB1 can bind to promoter region of Lin28A and Lin28B, which was detected by ChIP (I) and ChIP-qPCR (J). The effect of different concentration of EGF on the expression of miR-638 (K). The effect of different concentration of VASP expression was detected by qPCR (L) and western blot (M). The schematic diagram of the network of EGF/CREB1/Lin28/miR-638/VASP in breast cancer. *P < 0.05, **P < 0.01, ***P < 0.001.

To further analyze the mechanism of VASP in breast cancer, we investigated the crosstalk between the Transcription and post-Transcription regulation. The CREB1-related ChIP-Seq data showed that CREB1 was also enriched in the promoter region of Lin28A and Lin28B **(Figure 7A-B)**. Using MatInspector Professional, we found that there were a series of CREB1 potential binding sites identified within a 2-kb region upstream of the promoter region of Lin28A and Lin28B. Knockdown of CREB1 significantly inhibited the mRNA and protein expression levels of Lin28A and Lin28B **(Figure 7C-F)**. Then we constructed the Lin28A and Lin28B promoter reporter plasmids (pGL3-Lin28A-promoter, pGL3-Lin28B-promoter). The Luciferase assay showed that CREB1 overexpression significantly increased the activity of pGL3-Lin28A-promoter and pGL3-Lin28B-promoter **(Figure 7G-H)**. Chromatin immunoprecipitation and qPCR assays also showed that CREB1 can bind to the promoter regions of Lin28A and Lin28B **(Figure 7I-J)**. These results demonstrated that CREB1 acted as a transcription factor to activate the expression of Lin28A and Lin28B.

Binding of epidermal growth factor (EGF) to epidermal growth factor receptor (EGFR) leaded to the phosphorylation of CREB1 which resulted in a series of genes expression correlated with the progression of breast cancer. Our results showed that the treatment significantly inhibited the expression level of miR-638 and promoted the mRNA and protein expression levels of VASP through phosphorylation of CREB1 **(Figure K-M)**. Taken together, our results revealed that EGF can up-regulate the phosphorylation levels of CREB1 to increase the transcriptional level of VASP and Lin28A and Lin28B. Meantime, up-regulated Lin28A inhibited the maturation of miR-638, which released the inhibition of miR-638 on the post-transcriptional level of VASP, further promoting the expression of VASP protein **(Figure 7N)**.

## Discussion

The process of cancer metastasis comprises of a series of sequential steps including alterations in cell polarity and shape, which requires dramatic spatial and temporal reorganization of the actin cytoskeleton^18^. The mutations and abnormal expression of actin-associated proteins play an important role in the process via participating in regulation actin cytoskeleton inside cancer cells. Vasodilator-stimulated phosphoprotein (VASP) belongs to the Ena/VASP protein family and possesses actin binding and (anti-) capping activities, which facilitate to regulate actin polymerization and cytoskeleton dynamics by interacting with profilin and other actin regulatory factors 9. Studies have shown that the expression level of VASP in lung adenocarcinoma tissues is significantly higher than that in normal tissues, and the expression level of VASP is correlated with tumor differentiation 19. Increasing VASP expression in NIH3T3 fibroblasts may cause a tumorigenic phenotype 20, VASP silencing downregulated Migfilin, β-catenin and urokinase plasminogen activator, inhibited tumor spheroid invasion in MDA-MB-231 cells, and phosphorylation of VASP has been suggested as a marker for the potential of metastatic progression of prostate and breast tumors 21, 22. Our previous series of work showed that VASP was a key target protein for regulating the migration of various tumor cells, high expression of VASP was positively correlated with poor differentiation of gastric adenocarcinoma 11. Inhibition of VASP expression suppressed tumor cell adhesion and migration in cervical cancer, gastric cancer and breast cancer cells 12, 23. In addition, we found that the inflammatory factor TNF-α could inhibit breast cancer cell adhesion and proliferation via the HIF-1α/VASP pathway 13, 14. Berberine could inhibit the migration of breast cancer cells by targeting VASP and altering its spatial structure 24. These studies indicated that VASP could regulate cell migration and promote tumor invasion and metastasis. Although our previous series of studies have shown that VASP plays an important role in the development and progression of breast cancer, the role of VASP in breast cancer and its regulation mechanism remains to be further explored.

VASP is upregulated in breast cancer tissues and cells and is associated with proliferation and metastasis of breast cancer cells. Therefore, exploring the molecular mechanism of VASP upregulation in breast cancer cells may be a new strategy for targeted therapy of breast cancer. Transcriptional and post-transcriptional levels are the primary means of upregulation of gene expression levels. In the preliminary work of this group, we found that the protein expression level of VASP in breast cancer tissues was significantly higher than that in normal breast tissues. This suggested that in breast cancer tissues, VASP may have abnormal activation at the transcriptional and post-transcriptional levels. Therefore, we used bioinformatics methods to find transcription factors and miRNAs that may regulate VASP expression. Using bioinformatics software to screen miRNAs targeting VASP, we found that multiple miRNAs such as miR-638 and miR-610 could target the 3'UTR region of VASP. In our previous studies, it has been found that miR-610 can inhibit the migration and invasion of gastric cancer cells by targeting the 3'UTR region of VASP 15. In this study, we continue to explore whether there are other miRNAs that can regulate the expression of VASP. Among them, miR-638 expression level significantly decreased in breast cancer, non-small cell lung cancer and colon cancer and other tumors, and is closely related to tumor cell proliferation and invasion and metastasis. Next, *in vitro* study we found that miR-638 could target the 3' UTR of VASP and inhibited VASP expression at the post-transcriptional level.

The maturation process of miRNA mainly involves the transcription of the host gene into a primordial miRNA (pri-miRNA), which is recognized by the RNaseIII nuclease Drosha and processed to form a hairpin-like precursor miRNA (pre-miRNA) of about 65-70 bp 25, then the pre-miRNA is transported into the cytoplasm via a RanGTP/exportin 5-dependent manner, in which the pre-miRNA is recognized by Dicer and cleaved into a 17-25 bp double-stranded miRNA, followed by a helicase With the help of melting to form mature miRNAs 26, the recognition and processing of Drosha and Dicer are crucial for the formation of miRNA matures during the maturation process. The double-stranded RNA binding protein Pasha (partner of Drosha) can assist Drosha to recognize pri-miRNAs, inhibition of Pasha expression in Drosophila cells and nematodes may disrupt the cleavage of pri-miRNA, increases intracellular pri-miRNA accumulation, and reduces the amount of mature miRNAs 26. Lin28 is a highly conserved RNA-binding protein that recruits terminal uridylyltransferase (TUT4) by binding to the Lin28 recognition motif (GGAG) on the terminal loop structure of pre-let-7, promoting terminal uridine of pre-let-7, blocking Dicer's shearing of pre-let-7, thereby interfering with the processing maturation of let-7 27. In addition, since the precursor of miR-107 also contains this recognition motif, Lin28 can also inhibit their processing maturation 28. In the present study, we found that the stem region of pri-miR-628 contains the Lin28 recognition motif, which confirmed that Lin28 can affect the processing of miR-628, inhibit its maturation, reduce its expression level, and thus promote the expression of VASP.

Next, we used a variety of bioinformatics software such as Genomatix, JASPAR, to predict the transcription factors that may regulate VASP expression. The results of bioinformatics analysis showed that there were multiple potential transcription factor binding sites such as CREB1, HIF-1α, SMAD, and SP-1 in the promoter region of VASP. Our previous studies have found that HIF-1α can promote the proliferation of breast cancer cells by activating VASP 13, 14. CREB1 is a leucine zipper-type transcription factor that promotes transcription of target genes by recognizing CRE elements in the regulatory region of target gene 29. Through tissue, cell and molecular levels analysis, we found that CREB1 was abnormally highly expressed in breast cancer and positively correlated with VASP expression, and the promoter region of VASP contained two CRE elements, and overexpression or activation of CREB1 could significantly promote the expression of VASP, which suggested that CREB1 could act as a transcription factor to activate the expression of VASP, thereby promoting the proliferation and migration of breast cancer cells. In addition, we also found that there were multiple CRE sites in the promoter region of Lin28A and Lin28B, and CREB1 also regulated the expression of Lin28A and Lin28B as transcription factors. Our study suggested that CREB could directly regulate the expression of VASP, and could also regulate the expression of VASP through the Lin28/ miR-638 pathway.

In summary, we found that CREB1 regulated VASP expression in two ways in breast cancer. On the one hand, CREB1 acted as a transcription factor directly binding to the promoter of VASP and promotes VASP expression. On the other hand, CREB1 acted as a transcription factor to promote the expression of Lin28, affecting the processing of miR-628, inhibiting its maturation, reducing its expression level, thereby promoting the expression of VASP. Our study elucidated the mechanisms of the CREB1/Lin28/miR-638/VASP interactive network in the development of breast cancer, further explained the mechanism of VASP as a key oncogene, and provided new ideas for targeted therapy research against breast cancer cells growth and metastasis.

## Supplementary Material

Supplementary figures and tables.

## Abbreviations

VASP: Vasodilator-stimulated phosphoprotein
CREB1: cAMP responsive element binding protein 1
EGF: Epidermal growth factor
Lin28A: Lin28 Homolog A
Lin28B: Lin28 Homolog B
TUT4: Terminal uridylyltransferase
