# Supplementary material for: CREB1/Lin28/miR-638/VASP Interactive Network Drives the Development of Breast Cancer
Source: Int J Biol Sci. 2019 Oct 21;15(12):2733–49. doi: 10.7150/ijbs.36854 (PMC6854368; doi:10.7150/ijbs.36854)
Supplement: Supplementary file 1 — Supplementary figures and tables. [file ijbsv15p2733s1.pdf]

Supplementary Table

The primers of qPCR	
VASP	Forward: 5´-CTGGGAGAAGAACAGCACAACC-3´<br>Reverse: 5´-AGGTCCGAGTAATCACTGGAGC-3´
CREB1	Forward: 5´-CCCAGCCATCAGTTATTCAG-3´<br>Reverse: 5´-GAGTTGGCACCGTTACAGTG-3´
miR-638	Forward: 5´-AGGGATCGCGGGCGGGTGGCGGCCT-3´<br>Reverse: 5´-ATTCTAGAGGCCGAGGCGGCCGACATGT-3´
GAPDH	Forward: 5´-TGATGACATCAAGAAGGTGGTGAAG-3´<br>Reverse: 5´-TCCTTGGAGGCCATGTGGGCCAT-3´
Lin28A	Forward: 5´-GAGCATGCAGAAGCGCAGATCAAA-3´<br>Reverse: 5´-TATGGCTGATGCTCTGGCAGAAGT-3´
Lin28B	Forward: 5´-TCAGGTTGGGTTGCCAGTCCTTTA-3´<br>Reverse: 5´-TAAGCTTTGCATCACAGCACACCG-3´
**The primers of ChIP-PCR**	
VASP ChIP primer	Forward: 5´-CTGATTTCCCAGGGCTCG-3´<br>Reverse: 5´-AGGCTTCCGCAGCGTGTC-3´
Lin28A ChIP primer	Forward: 5´-TCGCATCACTTGAGAACC-3´<br>Reverse: 5´-GCTCCGTGTACCTCTGTTC-3´
Lin28B ChIP primer	Forward: 5´-ACAACTTGAAAGGCTGAT-3´<br>Reverse: 5´-TCACAGTCTTGCCTGATT-3´
**The primers of plasmids construction**	
pMIR-VASP-3' UTR	Forward:<br>5´-GGACTAGTCCCACAGGGACCCAGAAGACCC-3´<br>Reverse:<br>5´-CCCAAGCTTCTGCAGGGGCCCATTTTCAAG-3´
pMIR-VASP-3' UTR-Mut	Forward:<br>5´-CCCTTGGGGCCGGAGGGTCTGCTGGGGATGCACC-3´<br>Reverse:<br>5´-GGTGCATCCCCAGCAGACCCTCCGGCCCCAAGGG-3´
pcDNA-miR-638	Forward:<br>5´-CGCGGATCCGACGCCTTCAGCTCCATCGG-3´<br>Reverse:<br>5´-CCGCTCGAGTCCAGCGCACCTCCTGTCCTAC-3´
pcDNA-miR-638-Del	Forward:<br>5´-GGTGGCGGCCTAGGGCGCGGGGCGGACCGGGAATGGC-3´

	Reverse:<br>5′-GCCATTCCCGGTCCGCCCCGCGCCCTAGGCCGCCACC-3′
pcDNA-miR-638-Mut	Forward:<br>5′-GGTGGCGGCCTAGGGCGCTTCTGGCGGACCGGGAATGGC-3′<br>Reverse:<br>5′-GCCATTCCCGGTCCGCCAGAAGCGCCCTAGGCCGCCACC-3′
EX-Lin28A	Forward:<br>5′-GAAGATCTATGGGCTCCGTGTCCAACCAG-3′<br>Reverse:<br>5′-CGGGATCCTCAATTCTGTGCCTCCGGGAG-3′
pEGFP-CREB1	Forward:<br>5′-CGGAATTCCATGACCATGGAATCTGGAGCCGAGA-3′<br>Reverse:<br>5′-CGCGGATCCTTAATCTGATTTGTGGCAGTAAAGG-3′
pGL3-Lin28A-promoter	Forward:<br>5′-CGGGGTACCCTTCTACTTTGGTCTTCTTTGACTC-3′<br>Reverse:<br>5′-GGAAGATCTAATAGCGGTGGGAGGGCCCACGGCT-3′
pGL3-Lin28B-promoter	Forward:<br>5′-CGGGGTACCCCTTGCTTTTTCCCCCCAGTACATT-3′<br>Reverse:<br>5′-CCGCTCGAGACATGCTTTCTTGCTACTAATTTCA-3′
pGL3-VASP-748	Forward: 5′-TATGGTACCACTTCCTCCTCACCTTCCC-3′<br>Reverse: 5′-TATCTCGAGCATAGAGTCCGGCTTCCTG-3′
pGL3-VASP-1542	Forward:<br>5′-TATGGTACCGAAAAAAAGAAATTTAGTCTACC-3′<br>Reverse: 5′-TATCTCGAGCATAGAGTCCGGCTTCCTG-3′
pGL3-VASP-1542-mut	Forward:<br>5′-CTTGGCCTGGAAGGACTCAATGCTCCCCAGGTAAAG-3′<br>Reverse:<br>5′-CTTTACCTGGGGAGCATTGAGTCCTTCCAGGCCAAG-3′
pGL3-VASP-2100	Forward:<br>5′-TATGGTACCCTCCGGCATGGGCAACAAAG-3′<br>Reverse: 5′-TATCTCGAGCATAGAGTCCGGCTTCCTG-3′
